# A new mode of SAM domain mediated oligomerization observed in the CASKIN2 neuronal scaffolding protein

**DOI:** 10.1186/s12964-016-0140-3

**Published:** 2016-08-22

**Authors:** Ekaterina Smirnova, Jamie J. Kwan, Ryan Siu, Xin Gao, Georg Zoidl, Borries Demeler, Vivian Saridakis, Logan W. Donaldson

**Affiliations:** 1 Department of Biology, York University, 4700 Keele Street, Toronto, ON M3J 1P3 Canada; 2 Division of Computer, Computational Bioscience Research Center, Electrical and Mathematical Science and Engineering, King Abdullah University of Science and Technology, Thuwal, 23955-6900 Kingdom of Saudi Arabia; 3 Department of Psychology, York University, 4700 Keele Street, Toronto, ON M3J 1P3 Canada; 4 Department of Biochemistry, University of Texas Health Science Center at San Antonio, 7760 Floyd Curl Drive, San Antonio, TX 78229-3900 USA

**Keywords:** Analytical ultracentrifugation, Cell signaling, Crystal structure, Neuroscience, Nuclear magnetic resonance, Protein structure, Scaffold protein

## Abstract

**Background:**

CASKIN2 is a homolog of CASKIN1, a scaffolding protein that participates in a signaling network with CASK (calcium/calmodulin-dependent serine kinase). Despite a high level of homology between CASKIN2 and CASKIN1, CASKIN2 cannot bind CASK due to the absence of a CASK Interaction Domain and consequently, may have evolved undiscovered structural and functional distinctions.

**Results:**

We demonstrate that the crystal structure of the Sterile Alpha Motif (SAM) domain tandem (SAM1-SAM2) oligomer from CASKIN2 is different than CASKIN1, with the minimal repeating unit being a dimer, rather than a monomer. Analytical ultracentrifugation sedimentation velocity methods revealed differences in monomer/dimer equilibria across a range of concentrations and ionic strengths for the wild type CASKIN2 SAM tandem and a structure-directed double mutant that could not oligomerize. Further distinguishing CASKIN2 from CASKIN1, EGFP-tagged SAM tandem proteins expressed in Neuro2a cells produced punctae that were distinct both in shape and size.

**Conclusions:**

This study illustrates a new way in which neuronal SAM domains can assemble into large macromolecular assemblies that might concentrate and amplify synaptic responses.

**Electronic supplementary material:**

The online version of this article (doi:10.1186/s12964-016-0140-3) contains supplementary material, which is available to authorized users.

## Background

**Fig. 1:**
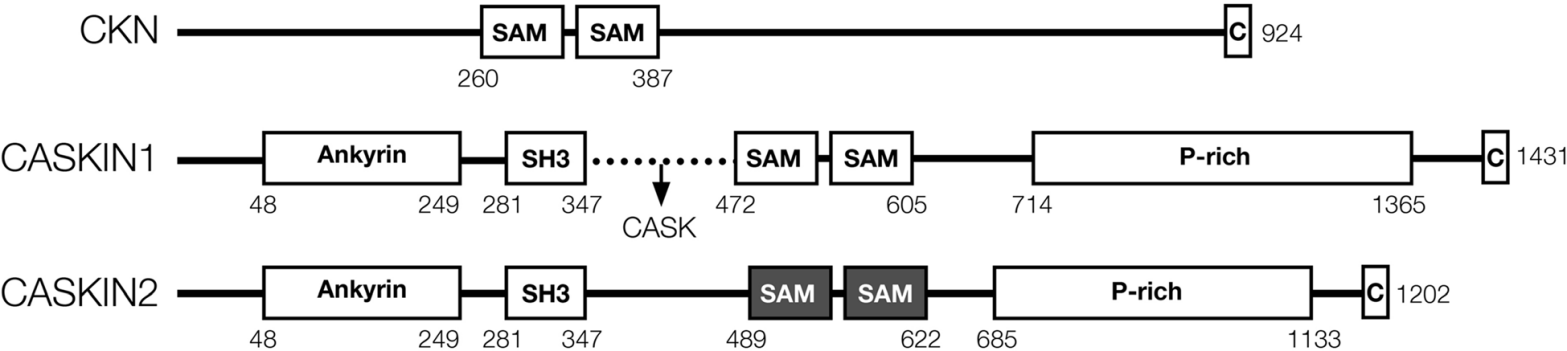
Conservation of the tandem SAM domains among three neuronal signaling scaffolding proteins, *Drosophila* Ckn, human CASKIN1, and human CASKIN2. The location of the binding site in CASKIN1 for the scaffolding protein CASK is shown by an arrow. The CASKIN2 SAM tandem described in this study is shaded *grey*

CASKIN2 and its mammalian homolog CASKIN1, are multidomain proteins that share the same overall organization [1]. The amino terminal half of both proteins consist of protein-protein interaction modules, namely six ankyrin repeats, an SH3 domain, and two SAM domains (Fig. 1). The carboxy terminal half consists of low complexity, proline-rich sequences [2] ending with a conserved 25 aa. segment of unknown function. The CASKINs are named for their ability to interact with CASK (calcium/calmodulin-dependent serine kinase), a MAGUK protein that is implicated in a number of neurological conditions including autism and X-linked mental retardation [3–7]. Only one homolog, *Ckn*, is observed in the *Drosophila* genome [8] and no homologs are observed in *C. elegans* suggesting that from an evolutionary perspective, multiple mammalian CASKINs may have arisen to promote a more comprehensive set of signaling circuits. In CASKIN1, the CASK interaction domain (CID) is located between the SH3 and SAM1 domains and facilitates direct contact with the calmodulin kinase catalytic domain of CASK. The CID is also present in the scaffolding protein, X11/Mint [9]. The CID, however, is not present in CASKIN2 rendering it unable to bind CASK [1]. Thus, despite their organizational similarity, CASKIN1 and CASKIN2 may have diverged with respect to their scaffolding functions in neurons, their structures and their protein partners.

Sterile Alpha Motif (SAM) domains are well represented in the human genome reflecting the versatility of this compact, five-helix fold to facilitate protein-ligand interactions that include other proteins, nucleic acids and lipids [10]. The most prevalent partners of SAM domains are, in fact, other SAM domains leading to a variety of homotypic and heterotypic SAM-SAM interactions in transcription factors [11, 12] and neuronal signaling protein assemblies [13–15]. Because SAM domains generally employ two complementary surfaces, homotypic interactions may produce not only dimers, but also assemblies of SAM domains polymers to highlight the considerable molecular weight they can attain [16].

The CASKIN1 and CASKIN2 tandem SAM domains were first identified to self-associate during an electron microscopy based survey which sought to identify new SAM domain mediated polymers [16]. Later high resolution X-ray studies revealed that the CASKIN1 SAM1-SAM2 tandem self-associated into helical fibrils [17]. Two roles have been proposed for this architecture at presynaptic sites. First, oligomers of CASKIN1 could link and concentrate cell-adhesion proteins including Ephrin B1 and CASK-associated neurexin. Second, oligomers of CASKIN1 could form a tether by which a stream of vesicles loaded with chemical transmitters could be guided via synaptogamin to the synaptic cleft [17].

We begin this report with a crystal structure demonstrating that the tandem SAM domains of CASKIN2 form an oligomer that is distinct from CASKIN1. By analytical ultracentrifugation, a dissociation constant describing the monomer-dimer equilibrium of the SAM tandem was observed to be in the micromolar range, a favorable concentration in the cell for tuning oligomerization and opening up the possibility for additional regulation by post-translational modifications and protein partners. An EGFP-tagged CASKIN2 SAM1-SAM2 protein expressed in neuroblastoma cells formed punctae consistent with high order oligomers while a structure-directed surface mutant was distributed diffusely. In support of the structural distinction between CASKIN1 and CASKIN2, the punctae were morphologically different. This study provides a foundation to begin exploring the effect of protein partnerships and post-translational modifications that direct the oligomeric state of CASKIN2 and consequently, its function in neurons, possibly apart from the processes directed by CASKIN1.

## Results

### The SAM domains of CASKIN2

**Fig. 2:**
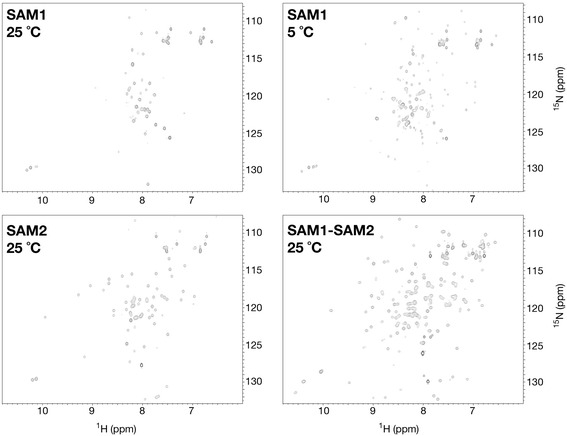
In isolation, CASKIN2 SAM1 and SAM2 demonstrate different thermostabilities*.* ^1^H-^15^N HSQC spectra acquired at 700 MHz at a protein concentration of 100 μM in PBS buffer supplemented with 10 % D_2_O. At 25 °C, SAM1 appears to be partially unfolded as the spectrum shows poor amide resonance dispersion as well as fewer resonances than expected. When the SAM1 sample is reacquired at 5 °C, more resonances are apparent. In contrast, the spectrum of SAM2 suggests that it is folded at 25 °C. The spectrum of the SAM1-SAM2 tandem is not an addition of the individual SAM1 and SAM2 spectra suggesting an interaction between the two domains

Prior to the structural studies, sequence alignments and secondary structure predictions were performed to define the boundaries of each five-helix SAM domain. These boundaries were experimentally established through the production of pure, ^15^N-labeled SAM1, SAM2 and SAM1-SAM2 proteins for NMR spectroscopy. At room temperature, SAM2 appeared to be folded due to the excellent dispersion and uniform resonance intensities observed in ^1^H,^15^N-HSQC spectra (Fig. 2). SAM1, however, demonstrated the spectral characteristics of a partially unfolded protein with fewer than expected resonances and limited chemical shift dispersion. Upon cooling the SAM1 protein to 5 °C and reacquiring spectra, a greater number of upfield and downfield resonances were observed suggesting that SAM1 was stabilized at low temperature. The ^1^H,^15^N-HSQC spectrum of SAM1-SAM2 was not the straightforward addition of the SAM1 and SAM2 spectra suggesting the two domains were coupled. Throughout the course of these studies, we noted that SAM1-SAM2 had a strong tendency to oligomerize as evidenced by increased spectral line widths at concentrations greater than 50 μM and was affected by temperature and ionic strength.

### Crystal structure of the SAM1-SAM2 tandem

Serendipitously, we observed microcrystal formation during the concentration of the CASKIN2 SAM1-SAM2 tandem preparations for NMR spectroscopy at high salt concentration (0.5 M NaCl). The salt dependence on crystallization was explored by a sparse matrix screen of crystallization conditions. The structure was subsequently solved at 2.75 Å resolution from a SAD dataset acquired at the Canadian Light Source Synchrotron (Table 1). A single SAM domain tandem was observed in the asymmetric unit. From a survey of the crystal contacts, the minimal biological unit was assigned to a dimer, which then repeated as a large oligomer.

**Table 1:** Data collection and refinement statistics

Data collection	
Space group	P6_5_22
Cell dimensions (Å)	96.4, 96.4, 119.2
Wavelength (Å)	0.97912
Reflections	377 578 (38 328)
Unique reflections	9004 (881)
Multiplicity	41.9 (43.5)
R-merge (%)	7.7 (0.86)
< I */ σ(I*)>	56.8 (4.68)
Completeness (%)	100.0
Refinement	
Resolution	48.51-2.75
Reflections	9004
*R*_*work*_	0.2449
*R*_*free*_	0.2649
Protein atoms	1100
Protein residues	140
Water molecules	0
RMSD bond lengths (Å)	0.012
RMSD angles (°)	1.269
Ramachandran statistics	
Most favored (%)	92.14
Additional allowed (%)	7.86
Disallowed (%)	0.0

Values in parentheses correspond to the highest resolution shell (2.85-2.75 Å)

**Fig. 3:**
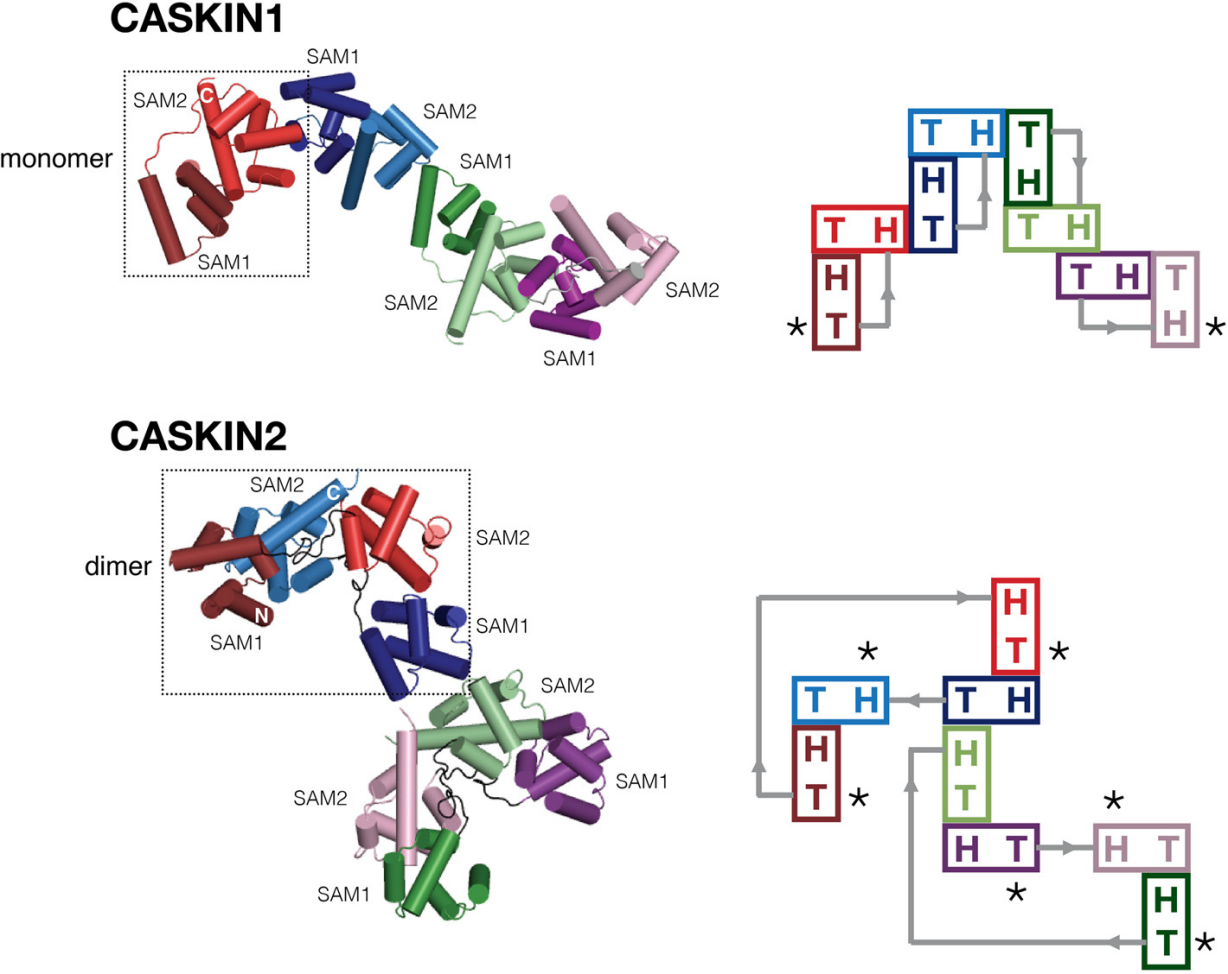
A comparison of the CASKIN1 (PDB: 3SEI) and CASKIN2 SAM domain tandem oligomers*.* Each SAM1-SAM2 tandem is colored individually, with SAM1 represented by a darker shade. The repeating unit is boxed. All of the intra- and intermolecular SAM domain interactions shown follow a head-to-tail type interaction. The head surface is derived from helices 2, 3 and 4 while the tail surface is predominantly derived from helix 5. To the right of each structure is a schematic illustrating the interactions between head and tail surfaces. An asterisk denotes available head and tail surfaces. Note that the CASKIN1 oligomer can only grow as a fibril in both directions while the CASKIN2 oligomer can form a branched structure

Despite having ~60 % sequence identity to CASKIN1 SAM tandem, we observed a different oligomeric architecture in the crystal structure of the CASKIN2 SAM tandem [17]. Since each SAM domain bears a complimentary head and tail surface, a tandem can interact with itself, as in the case of CASKIN1, to form a tight unit which we will call a compact monomer. The unoccupied head and tail surfaces, in turned, can link compact monomers in both directions to produce long fibrils (Fig. 3). In contrast, the CASKIN2 SAM tandem presented here forms a domain swapped dimer where SAM1 interacts with SAM2 of a second molecule and *vice versa*. Since each SAM domain in the dimer has an available interaction surface, the CASKIN2 SAM domain oligomer has the potential to form a branched oligomer in contrast to the linear assembly observed for CASKIN1 (Fig. 3).

The intra-SAM domain contacts within one dimer and inter-SAM domain contacts between dimers follow a head-to-tail type interaction that has been observed in many homo- and heterotypic SAM-SAM structures including, but not limited to AIDA-1 [18], ANKS3/ANK6 [19], Ste11/Ste50 [20], LEAFY [21], Liprin-α/Liprin-β [22], Ph/Scm [23], Shank3 [13], Ship2/EphA2 [24, 25], TEL [26], and Yan/Mae [11].

**Fig. 4:**
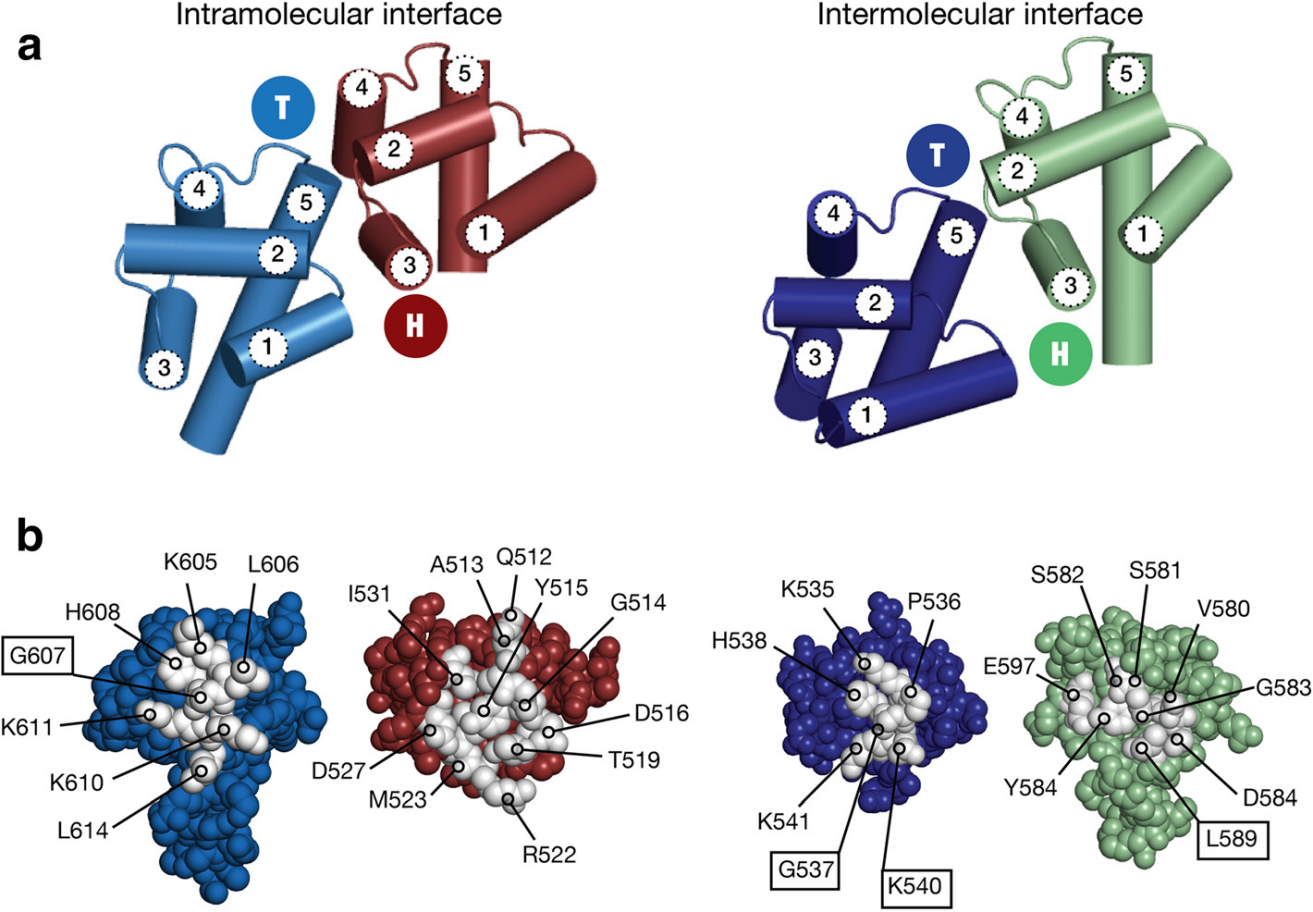
Detailed view of the complementary surfaces of the CASKIN2 SAM tandem, following the same color scheme as Fig. 3. **a** Cartoon representation of the five helices comprising each SAM domain, and the location of the complementary head (H) to tail (T) surfaces. The head surface is formed by contributions from helices 2, 3, and 4. The tail surface is formed by contributions mainly from helix 5. **b** Intermolecular contacts between at the intra- and intermolecular head and tail surfaces are labeled. Boxes indicated amino acids selected for mutagenesis

The head interaction surface of SAM2, located on the opposite side of this small globular domain, draws contributions from helices 2, 3, and 4. The tail interaction surface of SAM1 draws contributions nearly exclusively from helix 5. A detailed view of the head and tail surfaces of CASKIN2 SAM1 and SAM2 are presented in Fig. 4 and follow the same coloring scheme as Fig. 3, for clarity. While the SAM-SAM head-to-tail interaction is predominantly hydrophobic, ionic contacts serve an important role at the intramolecular SAM-SAM interface of the dimer and the intermolecular SAM-SAM interface between dimers. Specifically, ionic contacts were observed between D527/K610 and D516/K611 at the intramolecular SAM1-SAM2 interface and between H538/D585 and K540/D592 at the intermolecular SAM1-SAM2 interface. A more extensive ionic contact network was observed in the AIDA-1 neuronal scaffolding protein SAM tandem; a consequence of a highly basic nuclear localization signal being buried at the SAM-SAM interface. Ionic contacts also help the SHIP2 SAM domain discern its bona fide EphA1 and EphA2 SAM protein partners from other closely related SAM domains such as EphB2 [24].

**Fig. 5:**
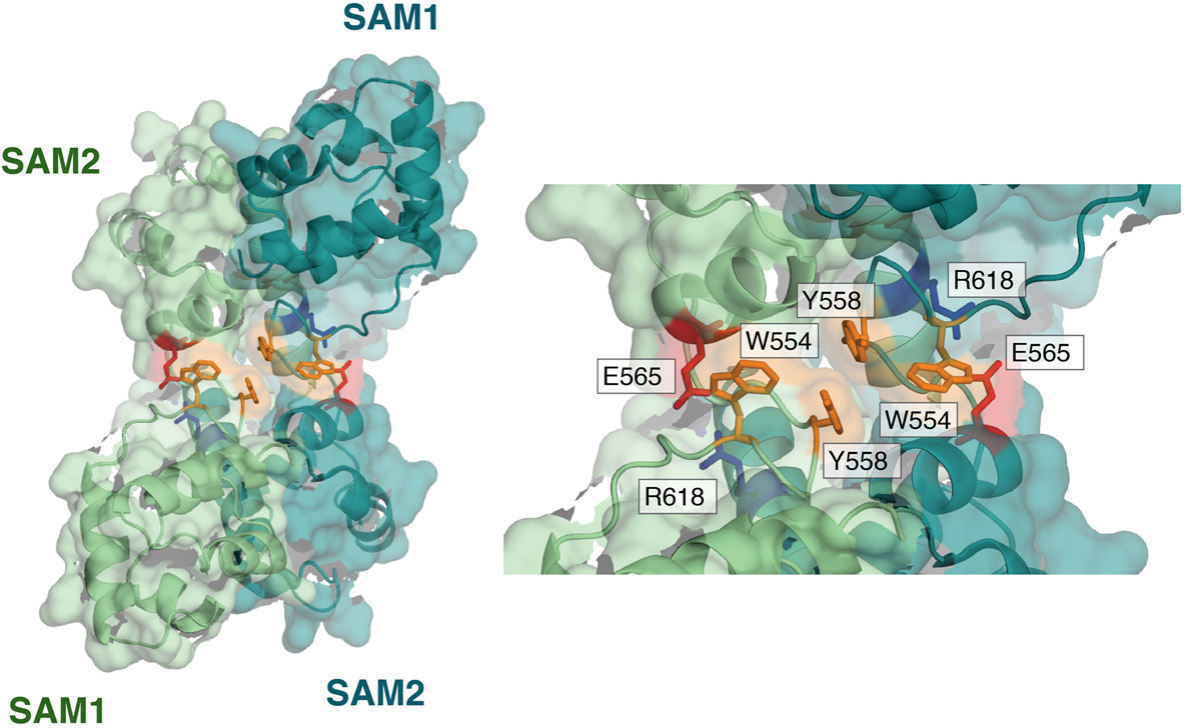
The linker interface in the CASKIN2 SAM tandem dimer. As observed in the crystal structure of the domain swapped dimer, SAM1 and SAM2 are restrained by intra- and intermolecular hydrophobic interactions between W554 and Y558 (*orange*) in the linker region. This central interface is further defined by an ionic interaction between E565 (*red*) and R618 (*blue*)

A hydrophobic network with contributions from W554 and Y558 and peripheral support from L555, together serve to restrain the linker in one conformation in the crystal structure (Fig. 5). These contacts, in turn, may limit the freedom that the two pairs of SAM domain have in solution. Near the linker, an ionic contact (E565/R618) from the SAM domains across the dimer interface further add to the compactness of the assembly. It is worth noting that in CASKIN1, Y558 is replaced by H542 and E565 is replaced by V549. Thus, both the hydrophobic and ionic contacts are not preserved in the linker and may contribute to the different types of oligomers observed. Finally, in this assessment of the linker region, we wish to emphasize that the sole conformation of the linker in the crystal structure should be interpreted with the caveats that it exhibited the highest B-factors in the refined model along with diminished electron density quality from an examination of an omit map that provides an unbiased assessment of the experimental data (Additional file 1: Figure S1).

To test if the two CASKIN2 SAM domains could bind each other independently, a ^1^H-^15^N HSQC reference spectrum of uniformly ^15^N-labeled SAM2 at 100 μM was initially acquired, followed by the addition of unlabeled (^14^N) SAM1 at a 1:1 stoichiometric ratio and reacquisition of the spectrum. From an examination of the overlaid spectra, only a few minor peak changes were observed in stark contrast to the spectrum of the tethered SAM1-SAM2 protein presented in Fig. 2. Thus, this experiment suggests that the two SAM domains must be tethered to interact with each other, with the linker potentially playing an active role in their association.

### Mutational analysis of the SAM domain interfaces

Consistent with the majority of SAM domain protein NMR and crystal structures solved to date, a head-to-tail type interaction facilitates SAM-SAM contacts within the dimer and throughout the oligomer. As a result, mutants on this surface can be designed that break one type of contact, intra- or intermolecular, while preserving the other. The substitution mutants described in this section are highlighted in Fig. 4.

The tail surface is comprised of residues from the beginning of helix 5. Within helix 5, a glycine plays a critical role because the absence of a side chain at this position permits the close approach of the helix backbone to the head surface of the opposing SAM domain. In CASKIN2 SAM1 and SAM2, these glycines are G537 and G607, respectively. According to the crystal structure, a substitution at G537 is predicted to preserve the dimer interface and inhibit oligomerization. Likewise, a substitution at G607 is predicted to decouple the SAM domains within the dimer leading to an open monomer similar to what was observed in the asymmetric unit of the crystal structure. Consistent with these predictions, an isotopically ^15^N-labeled G537D mutant was more soluble than the wild type SAM tandem and demonstrated an ^1^H-^15^N HSQC spectrum with excellent dispersion while an isotopically labeled G607D mutant demonstrated poor solubility and was only partially folded by a qualitative comparison of ^1^H-^15^N spectra with the wild type protein.

Using the same NMR survey employed for the G537D mutant, a modest increase in solubility was also observed for a K540E mutant. This substitution is located one helical turn down from the previously described G537D mutant. The combination of the two tail substitutions, expressed as a G537D/K540E double mutant, produced synergistic increase in solubility. This double mutant permitted solution NMR studies to be performed at a high protein concentration (0.8 mM, ~15 mg/mL) and temperature (37 °C). Furthermore, the favorable solution characteristics of the G537D/K540E double mutant made an interesting counterpoint to the wild type protein for additional in vitro and in vivo studies.

Given our success at breaking intermolecular contacts between dimers at the tail surface of SAM1, we also investigated an L589E mutant that was predicted to break contacts between dimers at the head surface of SAM2. ^1^H-^15^N HSQC spectra of isotopically labeled preparations of the L589E mutant presented the characteristic spectral dispersion of a folded and coupled SAM tandem but suffered from limited solubility similar to what we observed for the individual G537D and K540E mutants.

### Structural features of the G537D/K540E double mutant

**Fig. 6:**
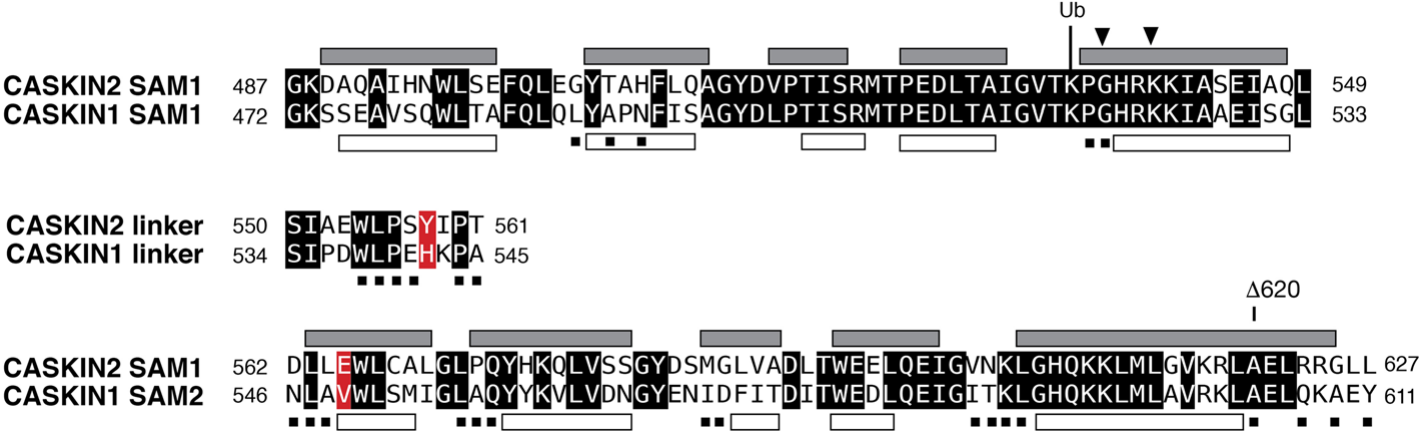
Secondary structure of the CASKIN2 SAM tandem by NMR and X-ray methods. Closed and open rectangles denote the five helices in each SAM domain. Triangles denote two surface exposed amino acids (G537, K540) whose substitution suppressed oligomerization. *Black* squares denote amino acids that could not be assigned in 950 MHz NMR spectra of a CASKIN2 G537D/K540E double mutant. Ub denotes a ubiquitin site observed from a global proteomics survey of CASKIN1 [36]. Δ620 identifies the site of a C-terminal truncation of the G537D/K540E double mutant to delimit the boundary of helix 5 in solution. In the sequence comparison with CASKIN2, *black* boxes denote sequence similarity. Two *red* boxes denote differences between CASKIN2 and CASKIN2 that are predicted to reduce hydrophobic and ionic contacts at, and in the vicinity of, the linker region

Since the G537D/K540E mutant was very soluble, a uniformly ^15^N/^13^C labeled sample was produced and studied by NMR methods. We had confidence that the SAM domain interactions were preserved because the ^1^H-^15^N HSQC spectra of the double mutant (at 0.8 mM) and wild type protein (at 0.01 mM) were superimposable (Supplementary Figure S2). From a set of conventional heteronuclear experiments acquired at 950 MHz, some backbone (HN, CA, CB, C) chemical shift assignments could not be made likely due to hydrogen exchange occurring at 37 °C and pH 7.8 (Fig. 6). Six of the thirteen backbone amide resonances in the linker region between the SAM domains could not be assigned suggesting it could be experiencing motions in the intermediate (μs-ms) timescale exacerbated by hydrogen exchange. Thus the data in solution appear to suggest that the linker in the G537D/K540E mutant is more flexible in contrast to the single conformation that observed in the wild type SAM tandem crystal structure.

In the crystal structure, the C-terminal segment of helix 5 in SAM2 extends to G658 and makes contacts with a similar segment in another dimer. From the solution NMR studies of the G537D/K540E double mutant that suppresses oligomerization, helix 5 is shorter, ending instead at L652 as evidenced by the absence of strong sequential backbone HN(*i*,*i + 1*) NOEs from this position onwards. Building upon the G537D/K540E framework, a ∆620 C-terminal deletion mutant was expressed and ^15^N uniformly labeled. The ^1^H-^15^N HSQC of this deletion mutant was virtually indistinguishable from the parent G537D/K540E confirming that helix 5 is not only shorter in the oligomerization-suppressed double mutant, the region from residue 620 onwards does not make any significant contributions to the SAM1-SAM2 fold.

A series of ^15^N T_1_ and T_2_ relaxation rate measurements were made on a ^13^C,^15^N labeled sample of the G537D/K540E double mutant at high concentration (0.8 mM) at 25 °C. From the spectra, 49 non-overlapping resonances corresponding to structured regions of protein were selected for further analysis with an average ^15^N T_1_ and T_2_ rates of 1.55 ± 0.15 s and 0.064 ± 0.002 s, respectively. From the T_1_/T_2_ ratios of each observation, an average correlation time of 13.2 ± 1.1 ns was determined. In terms of molecular weight, this correlation time corresponds to an isotropically tumbling protein of 29 ± 2 kDa. To put this observation in context, the monomeric molecular weight of the 6xHis tagged SAM1-SAM2 tandem is 20.4 kDa, and if the unstructured amino- and carboxy termini are ignored, the remaining 140 aa. contribute 15.7 kDa. Thus, the correlation time suggests that the SAM tandem in solution has characteristics of a protein assembly larger than a monomer, upwards to a dimer.

### Monomer-dimer equilibria of the wild type SAM tandem and an oligomerization suppressed double mutant

The oligomerization state of the wild type CASKIN2 SAM tandem is affected by temperature, protein concentration and ionic strength. In our early NMR studies, a transition to the oligomer occurred at concentrations near the practical limit of the technique (~50 μM) leading us to pursue a structure-directed G537D/K540E double mutant that was resistant to oligomerization. However, we observed differences in the linker and helix 5 of SAM2 leading us to consider the possibility that the mutations affected the equilibrium between the monomeric and dimeric states. A solution of the G537D/K540E double mutant structure was not pursued because the observed correlation time suggested that there could be two indistinguishable states — a compact monomer similar to CASKIN1 crystal structure [17] and a dimer similar to the CASKIN2 crystal structure presented in this report.

To complement and extend these initial observations at high concentrations, a series of analytical ultracentrifugation/sedimentation velocity (AUC-SV) experiments were performed at two low concentrations (10 μM and 34 μM) and two ionic strengths (150 and 300 mM NaCl). AUC-SV is particularly well suited for studying mass action driven reversible associations and detecting subtle changes in thermodynamic behavior.

**Fig. 7:**
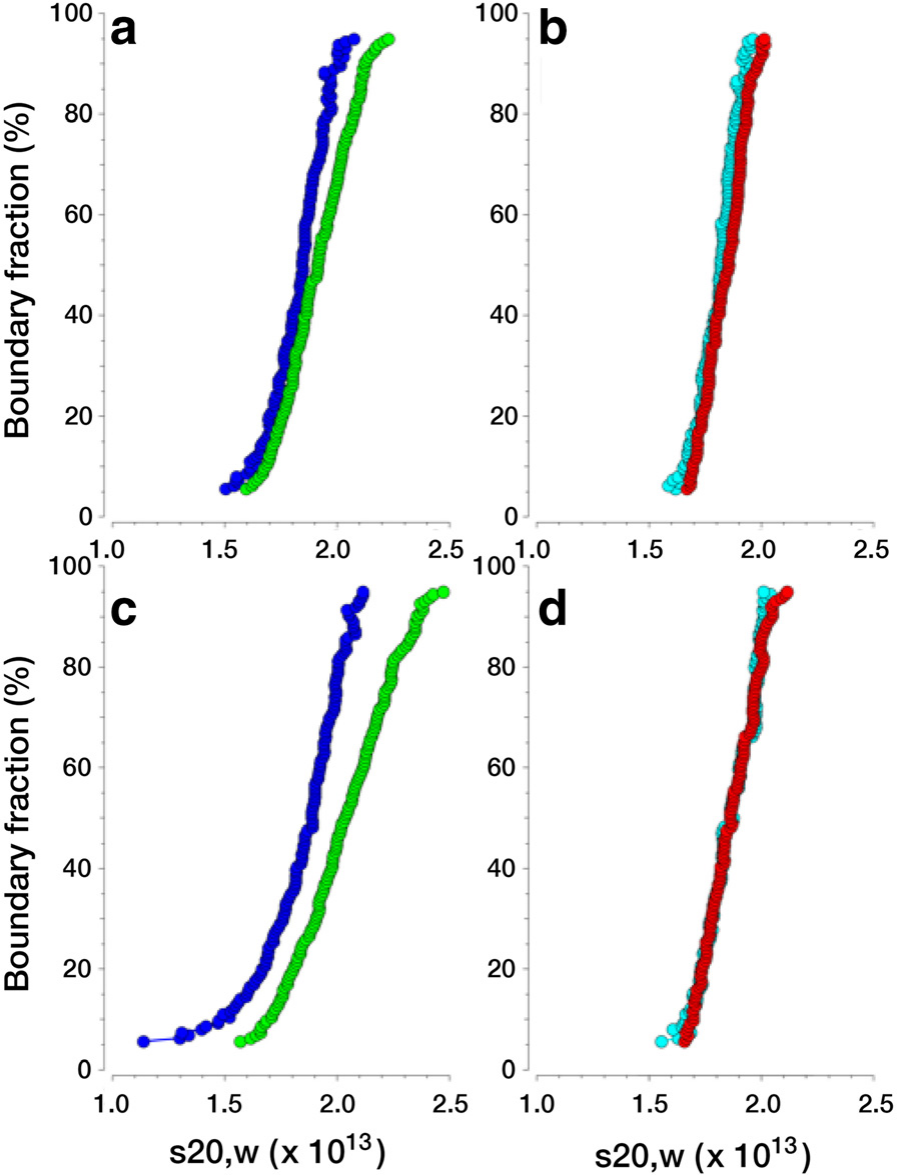
Van Holde - Weischet integral G(s) sedimentation coefficient distributions for CASKIN2 at 10 μM (wild type, *blue*; G537D/K540E double mutant, *cyan*) and 34 μM (wild type, *green*; G537D/K540E double mutant, *red*) loading concentrations. Panels **a** and **b** were measured at 150 mM NaCl, while panels **c** and **d** were measured at 300 mM NaCl. A shift in sedimentation coefficient for higher concentrations indicates reversible mass action. This effect is only seen for the wild type, not for the double mutant. Furthermore, the effect is enhanced at higher ionic strength, indicative of a decrease in *K*_*d*_ for the wild type. These results indicate that the double mutant only exists in a monomeric form at low concentration, while the wild type SAM tandem dimerizes and is more sensitive to changes in ionic strength

Representative diffusion corrected sedimentation profiles shown in Fig. 7 clearly demonstrate that the wild type CASKIN2 SAM tandem responds to mass action, while the G537D/K540E double mutant does not. In other words, as the concentration increases, oligomerization of the wild type SAM tandem increases and the diffusion corrected sedimentation distributions shift towards higher values. As we initially observed during crystallization trials, salt concentration was also observed to promote oligomerization in the analytical ultracentrifuge, with the highest protein and salt concentrations producing an additive effect. Quantitative *K*_*d*_ values and anisotropy information were obtained by fitting AUC-SV experiments from the 34 μM experiments to reversibly self-associating monomer-dimer equilibrium models using a genetic algorithm [27]. Only the higher concentration experiments were fitted, since these experiments cover a larger concentration range and therefore contain more signal, covering both monomer and dimer species with higher confidence. Since AUC-SV experiments produce a moving boundary which extends from zero concentration to the loading concentration (34 μM, in this case), reversibly self-associating systems will display a reaction boundary where the ratio of monomer to oligomer changes from 100 % monomer near zero concentration towards increasing amounts of the oligomeric species at the higher loading concentration. Fitting the entire reacting boundary shape with finite element solutions of the Lamm equation for reacting systems [28] then permits an accurate determination of the equilibrium constant. All AUC-SV experiments produced excellent fits with RMSD values comparable to the more degenerate 2DSA fits. The *K*_*d*_ values for all four measurements are summarized in Table 2. These results clearly show that the *K*_*d*_ determined for the double mutant far exceeded the loading concentration, suggesting essentially monomeric composition. The *K*_*d*_ of the double mutant at 300 mM NaCl concentration is approximately three-fold higher than the loading concentration, indicating that even under high salt conditions there is only negligible self-association. Thus, the AUC-SV study provides convincing evidence that the oligomerization deficient mutant G537D/K540E at a 34 μM concentration and below is a compact monomer.

**Table 2:** Monomer-dimer equilibrium constants for wild type CASKIN2 SAM1-SAM2 and an oligomerization-inhibited double (G537D/K540E) at two NaCl concentrations

	150 mM NaCl	300 mM NaCl
Wild type		
*K*_*d*_ (μM)	52.9 (49.3, 56.5)	27.6 (26.8, 28.3)
φ (monomer)	1.25	1.27
φ (dimer)	1.28	1.25
G537D/K540E		
*K*_*d*_ (μM)	n.d.	99.8 (98.9,100.7)
φ (monomer)	1.14	1.02

Values in parentheses represent the 95 % confidence intervals obtained from a genetic-algorithm Monte Carlo analysis. φ represents the anisotropy of the molecule, with a value close to 1.0 indicating a more compact and globular structure, while increasingly larger values reflect more extended shapes. Since the dimer concentration of the G537D/K540E mutant is negligible, the anisotropy of the dimer was not calculated. A *K*_*d*_ for the G537D/K540E mutant at 150 mM NaCl could not be detected (n.d.) because the sample was essential monomeric

A comparison of the anisotropy values from the AUC-SV analysis indicate that the monomeric and dimeric forms of the wild type SAM tandem are similarly compact (Table 2). The anisotropy values also indicated that G537D/K540E double mutant monomer is slightly more compact than the wild type SAM tandem reinforcing the observations from the NMR investigation that the double mutant and wild type SAM tandems have structural differences.

### Expression of the CASKIN1 and CASKIN2 SAM domain tandems in Neuro2a cells

**Fig. 8:**
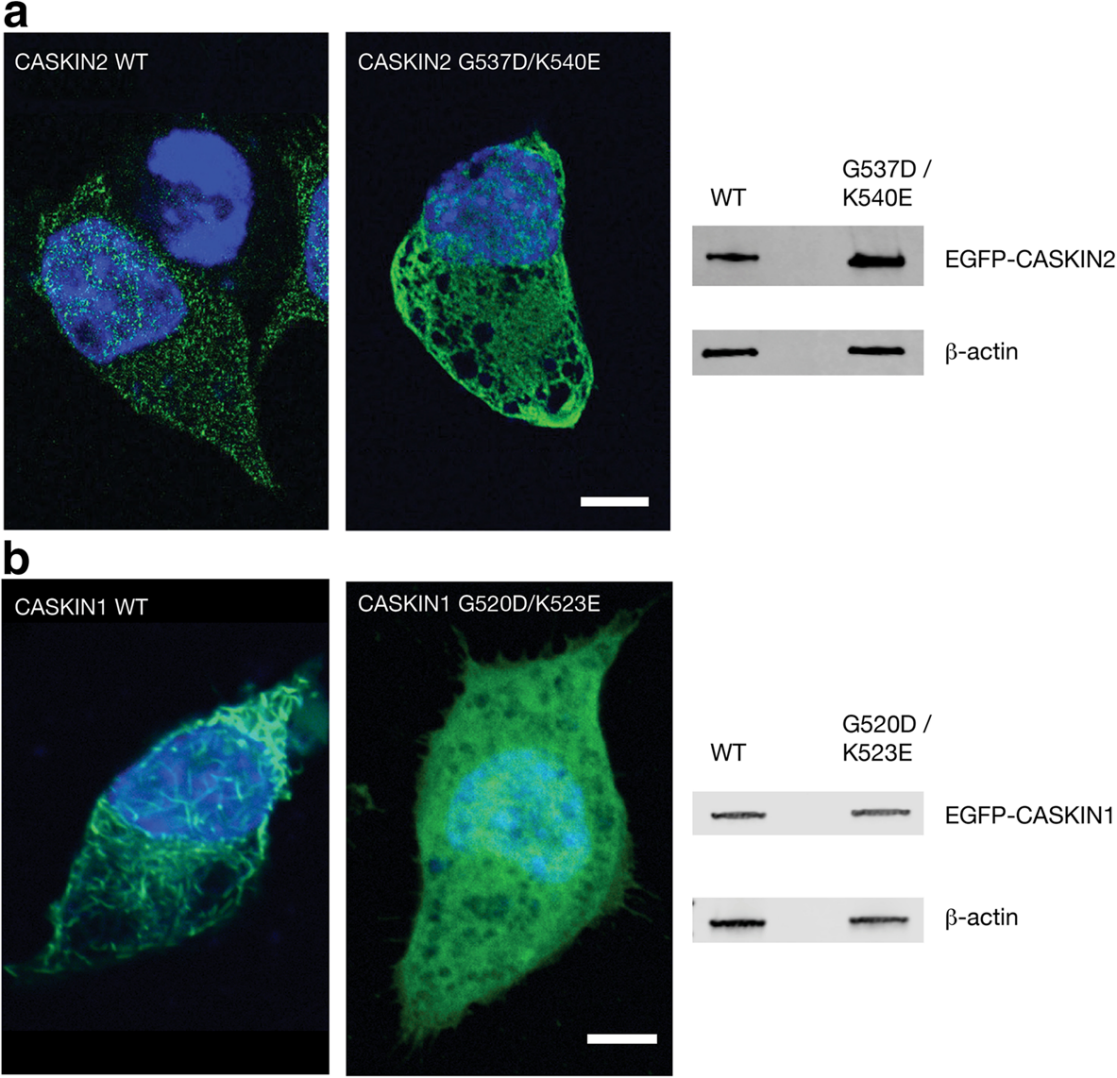
CASKIN2 and CASKIN1 SAM domain expression in Neuro2a cells. **a** Images were made 48 h after transient transfection with CASKIN2-EGFP (wild type), CASKIN1-EGFP (wild type), mutant CASKIN2 (G537D/K540E)-EGFP, and CASKIN1 (G520D/K523E)-EGFP plasmids. The *green* fluorescence demonstrates distinct protein distributions for the wild type and mutant proteins. Counterstaining with DAPI (*blue*) reveals that the subcellular distribution of wild type and mutant proteins in the cytoplasm and nucleus is indistinguishable. Scale bar: 5 μm. **b** Western blot of cell lysates demonstrating expression of EGFP-CASKIN2 and EGFP-CASKIN1 proteins probed with monoclonal anti-EGFP antibodies. The blot was reprobed with monoclonal anti-β-actin antibodies as a loading control

To begin understanding how oligomerization may affect cellular processes, we transfected EGFP fusions of wild type CASKIN2 SAM tandem (EGFP-WT) and the non-oligomerizing G537D/K540E mutant (EGFP-G537D/K540E) into Neuro2a cells. We chose to express only the SAM tandems to visualize their effect independently from the other protein interaction domains (ankyrin and SH3) in the amino terminal region and other unknown interaction motifs in the carboxy terminal region of CASKIN2. From a series of micrographs analyzed, one representative set is shown in Fig. 8a. Both EGFP-WT and EGFP-G537D/K540E were observed throughout the cell, including the nucleus. Nuclear localization by diffusion is possible since the molecular weight of the EGFP-CASKIN2 SAM tandem is ~50 kDa. While the fluorescence distribution was relatively uniform for the G537D/K540E mutant, fluorescence was concentrated in dense punctae for the wild type protein.

The same expression assay under similar conditions was performed with the EGFP-tagged CASKIN1 SAM tandem and its and an analogous double mutant G520D/K523E to the CASKIN2 G537D/K540E double mutant features in this report (Fig. 8b). While we did not perform an in vitro study to confirm that the CASKIN1 mutant was oligomerization-suppressed, it is worth noting that a CASKIN1 G520E single mutant described in [17] was sufficient on its own. Consistent with our observations for the CASKIN2 SAM tandem, the CASKIN1 SAM tandem double mutant was distributed throughout the cytoplasm and nucleus, and the wild type CASKIN1 SAM tandem formed punctae. The punctae, however, were distinct from the CASKIN2 SAM tandem, appearing not as condensed speckles, but rod-like structures throughout the cell. In summary, this in vivo expression study is consistent with our observations from crystallography — the CASKIN2 and CASKIN1 SAM domains self-associate differently and consequently present a different oligomeric architecture. The differences in the morphology of the aggregates cannot be explained by variances in concentration since both SAM tandems were expressed at approximately the same levels as an actin control.

## Discussion

We have presented data from a set of complimentary sources (X-ray crystallography, NMR spectroscopy, analytical ultracentrifugation, and in vivo expression) demonstrating that the CASKIN2 SAM tandem experiences concentration and salt dependent oligomerization. While the CASKIN2 SAM domain crystal structure presents a series of head-to-tail contacts that are typical for most self-associating SAM domains, the manner in which the oligomer is organized as a repeating set dimers is new and distinct from CASKIN1.

Analysis of the AUC-SV data suggests that the wild type CASKIN2 SAM tandem is in a reversible monomer-dimer equilibrium at low concentrations (10–34 μM). In contrast, the oligomerization suppressed G537D/K540E double mutant is essentially monomeric with the dimeric form only beginning to become apparent at high (>500 μM) concentrations. This difference between the wild type and double mutant proteins is qualitatively apparent in the magnitude of the shifts and shapes in sedimentation distributions. From these data and prior knowledge of the system, a quantitative approach using discrete reversible monomer-dimer equilibrium models were justified to determine a *K*_*d*_ of the wild type SAM tandem from the SV data directly at two ionic strengths. Consistent with the crystallization conditions, ionic strength enhanced dimerization for the wild type SAM tandem and to a much lesser extent for the G537D/K540E double mutant.

**Fig. 9:**
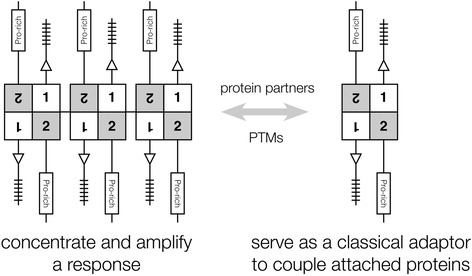
Signaling consequences of dimerization and oligomerization by the tandem SAM domains of CASKIN2. In its oligomeric form, CASKIN2 would provide a vast array of interaction sites with enhanced avidity for many proteins through its available intrinsically unstructured regions and ankyrin repeats. In its dimeric form, CASKIN2 could fulfill a classic adaptor role bringing together protein partners that potentially depend on dimerization themselves for coupling and activation

The *K*_*d*_ of the CASKIN2 SAM tandem is well suited to the anticipated levels of the protein at synaptic sites and within the realm of other signaling domains such as SH3 and WW domain that must rapidly sample ligands to fulfill their biological functions. At low concentrations, CASKIN2 in its monomeric or dimeric form could serve as a classical adaptor bringing protein partners together (Fig. 9). Furthermore, dimeric CASKIN2 may help activate associated proteins that depend upon dimerization. At higher concentrations, oligomeric CASKIN2 could provide the increased avidity to concentrate and amplify low affinity protein partnerships that would otherwise be suppressed.

Along with concentration, ionic strength can contribute to oligomerization, although it is unclear if cation fluxes associated with neuronal signaling are sufficient to serve a regulatory role. Pursuing this idea, we did observe a series of hydrophobic contacts in the central portion of the linker region supported by ionic contacts from the nearby SAM domains. In an analogous interaction mode to CASKIN2 domain-swapped dimer, hydrophobic interactions dominate in Byr2-SAM/Ste4-SAM [29, 30] and Ste11/Ste50 [20] heterodimer interface with peripheral support from charged/polar residues. Likewise, Ph [31], TEL [26] and Yan [11] homo-oligomers and Ph/Scm [23] hetero-oligomers assemble around a central hydrophobic cluster in the central head-tail interface supported by number of peripheral electrostatic interactions. An examination of the CASKIN2 and CASKIN1 sequences suggests that these contacts would not be preserved thereby providing a possible explanation for why the minimal repeating unit of CASKIN1 is a compact monomer while the minimal repeating unit of CASKIN2 is a dimer. Furthermore, since ionic contacts are involved, salt concentration and pH may also serve a role at mediating CASKIN2 dimerization and oligomerization. Indeed, as salt concentration increases, the *K*_d_ describing the monomer-dimer equilibrium increases. The precise effects of salt concentration could be complex as charges are screened and hydrophobic effects begin to predominate. The high concentration of sodium formate used to promote crystallization represents the extreme effect where the protein is essentially salted out of solution. While our investigation was limited to only two NaCl concentrations (0.15 and 0.3 M) and one pH, we refer the reader to a survey of the EphA2/SHIP2 SAM domain heterodimer for a comprehensive perspective of ionic interactions using NMR methods and molecular modeling [24].

EGFP-tagged CASKIN2 SAM1-SAM2 protein was observed as punctae when expressed in Neuro2a cells. This distinctive pattern is very similar to what has been reported for the oligomeric form of the Dishevelled DIX domain [32, 33]. Although they were less apparent, punctae were also observed in micrographs of GFP-tagged diacylglyercol kinase d1 (DGKδ1), facilitated by the Zn(II) dependent oligomerization of its single SAM domain [15, 34]. Mutations in the DGKδ1SAM domain that either abolished Zn(II) binding or inhibited oligomerization resulted in disappearance of punctae and translocation of DGKδ1 to the plasma membrane. Supplementing the structural study of the CASKIN1 SAM tandem, transfections of GFP-tagged full length CASKIN1 were performed in HEK293 cells with the majority of fluorescence observed in the cytoplasm along with some higher intensity speckles near the nucleus [17]. To enable a consistent comparison with the results presented in this study, the CASKIN1 SAM domain tandem and oligomerization inhibited double mutant were expressed in Neuro2a cells under similar conditions (vector, fluorescent reporter, protein levels) as the CASKIN2 SAM tandem. As shown in the micrographs, there was a striking difference in the morphology of the aggregates. Taken together with the crystal structures, the CASKIN2 and CASKIN1 SAM domains appear to oligomerize differently in vitro and in vivo. The biological consequences of this difference may reflect the divergent roles that each protein plays in the neuron.

If CASKIN2 oligomerization is an essential aspect of its neuronal signaling function, it stands to reason that there should be ways to regulate oligomerization that supersede solution conditions such as protein concentration, pH, and divalent ion concentration [34, 35]. Post-translational modifications and protein partner binding [11] offer targeted opportunities to affect the oligomerization process, by repressing the formation of oligomers or by facilitating the disassembly of oligomers. While no biological process that regulates CASKIN2 has been identified to date, a global mass spectrometry survey identified a ubiquitinated lysine (K536) in CASKIN1 at the same oligomerization surface as the G537D/K540E mutants described in this report [36]. Ubiquitination may possibly block the formation of oligomers in an analogous manner that has been reported for the *Dishevelled* DIX domain [37] and incorporate this neuronal signaling scaffolding protein into other signaling and translation pathways in the neuron.

## Conclusions

Sterile alpha motif (SAM) domains are versatile protein-protein interaction modules. Using the CASKIN2 scaffolding protein as a focus of this investigation, we have demonstrated that its SAM domain tandem is able to sample monomeric, dimeric, and oligomeric states. Given the structural distinctiveness of these states, CASKIN2 has the potential to support many different functions in neuronal signaling circuits.

## Methods

### Cloning

The human CASKIN2 SAM1-SAM2 tandem (483-634; Uniprot Q8WXE0) and individual SAM1 (483-549) and SAM2 (550-634) domains were amplified by PCR from a human cDNA and inserted into the *BamHI*/*XhoI* restriction sites of pET28 (Novagen) followed by transformation into *E. coli* BL21:DE3 to produce a 6xHis tagged protein. Five CASKIN2 mutants, G537D, K540E, G537D/K540E, L589E, and G607D were made using the Quikchange method (Agilent). An EGFP fusion protein to the wild type CASKIN2 SAM1-SAM2 tandem and G537D/K540E mutant was prepared by inserting a suitable PCR product into the *XhoI*/*KpnI* restriction sites of pEGFP-N1 (Clontech). A similar approach was used to make EGPF-tagged CASKIN1 SAM1-SAM2 (470-613; Uniprot Q8WXE9) and a G520D/K523E mutant using a synthetic CASKIN1 gene fragment (GenScript).

### Expression and protein purification

Isotopic labeling of CASKIN2 SAM1, SAM2, and SAM1-SAM2 for NMR spectroscopy was achieved by a 1.0 L fermentation in a minimal medium containing 1 g ^15^NH_4_Cl as the sole nitrogen source and/or 3 g of ^13^C-glucose as the sole carbon source. Proteins for X-ray crystallographic studies were expressed in a minimal medium with the addition of 50 mg/L of selenomethionine 15 min before induction. Cell pellets were dissolved in T300 buffer (20 mM Tris-HCl, 300 mM NaCl, 0.05 % NaN_3_) and lysed by French press. Highly purified protein was obtained from a two step purification involving Nickel-NTA affinity chromatography (Qiagen), followed by gel filtration chromatography (Sephacryl-100, HiLoad 16/60; GE Life Sciences). The final buffer for NMR analyses was phosphate buffered saline (PBS; 20 mM sodium phosphate, pH 7.8, 0.15 M NaCl, 0.05 % (w/v) NaN_3_. Crystallographic screening was performed with proteins in T300 buffer.

### Cell culture, transient transfection and immunoblotting

Neuroblastoma 2a (Neuro2a) cells [38] were maintained using standard growth conditions and used for expression and localization studies as described in [39]. 30,000 cells were seeded onto 13 mm glass cover slips in 24 well plates and 200–400 ng plasmid DNA transfected using Effectene reagent as recommended by the manufacturer (Qiagen). Whole cell protein lysates from transfected Neuro2a cells collected 48 h post-transfection were separated by 10 % SDS-PAGE and transferred to 0.2 μm Hybond-ECL nitrocellulose membrane (GE Life Sciences) for immunodetection. Primary antibodies were diluted 1:1000 (rabbit anti-GFP; Santa Cruz) and 1:20000 (mouse anti-β-actin; Sigma-Aldrich). Secondary antibodies (LI-COR Biosciences) were diluted 1:20000 (donkey anti-rabbit IRDye680LT) or 1:20000 (goat anti-mouse IRDye800CW). Signals were detected using the Odyssey Infrared Imaging System (LI-COR Biosciences).

### Confocal microscopy

Transfected cells were fixed with 4 % paraformaldehyde for 20 min at room temperature, washed with PBS, counterstained with DAPI and mounted for imaging. Samples were visualized using a Zeiss LSM 700 confocal microscope with a Plan-Apochromat 63x/1.4 Oil DIC M27 objective and the ZEN 2010 program to control all hardware parameters. Images were collected by line averaging (4x) at high resolution (2048x2048 pixel) using single planes or *z*-stacks. Images were exported and further processed using ImageJ. For deconvolution, the point-spread function was calculated using the Gaussian PSF 3D and Iterative 3D Deconvolve software plugins in ImageJ. Images were combined in Adobe Photoshop for presentation.

### Analytical ultracentrifugation

Sedimentation velocity (SV) experiments were performed with a Beckman Optima XL-I at the Center for Analytical Ultracentrifugation of Macromolecular Assemblies at the University of Texas Health Science Center at San Antonio. SV data were analyzed with UltraScan-III [40] All calculations were performed on the XSEDE UltraScan Science Gateway using high-performance computing resources at the Texas Advanced Computing Center, at the San Diego Supercomputing Center, and at the Bioinformatics Core Facility at the University of Texas Health Science Center at San Antonio. All measurements were made in 20 mM sodium phosphate buffer, pH 7.8, supplemented with 0.15 M or 0.3 mM NaCl. The experimental data were collected in intensity mode at 20 °C, and at 50,000 rpm, using standard epon-charcoal two-channel centerpieces. Hydrodynamic corrections for buffer density, viscosity and partial specific volume were made as implemented in UltraScan-III, except when equilibrium constants were fitted to whole boundary models. In those cases, the monomer molar mass, which is known, was held constant, and the partial specific volume was floated to account for the variability in partial specific volume under different salt concentrations. The experimental data were first modeled with solutions of the Lamm equation [41], which are fitted to experimental data by two-dimensional spectrum analysis [42] using meniscus fitting and simultaneous time- and radially invariant noise removal [43]. Noise corrected data were further analyzed by the enhanced van Holde - Weischet method [44]. This approach provides diffusion corrected sedimentation coefficient distributions, providing clear evidence for the presence of heterogeneity, and for identifying reversible mass action reactions. Quantitative equilibrium constants were obtained by fitting analytical ultracentrifugation sedimentation velocity (AUC-SV) experiments by genetic algorithm analysis of as described in [27]. Ninety-five percent confidence intervals were determined by Monte Carlo analysis [45].

### NMR spectroscopy

All experiments were performed with either uniformly ^15^N-labeled, or ^13^C,^15^N-labeled samples, as required. Assignment of the G537D/K540E mutant at 0.8 mM in was achieved by a conventional triple resonance strategy (HNCACB, CBCACONH, HNCO, HNCACO) acquired at 310 K with non-linear sampling on a Bruker Avance 950 MHz NMR spectrometer at the Imaging and Characterization Core Laboratory (KAUST). Datasets were processed with a combination of NMRpipe [46] and istHMS [47] and interpreted with CCPN Analysis [48]. Backbone ^15^N relaxation experiments at a protein concentration of 0.3 mM were acquired on a Bruker Avance 700 MHz NMR spectrometer at the York University Life Sciences Building Central Facility. A longitudinal ^15^N T_1_ relaxation rate was determined by acquiring 2D spectra with delays of 200, 400, 600, 800, 1000, and 1200 ms. A transverse ^15^N T_2_ relaxation rate was determined by acquiring 2D spectra with delays of 17, 34, 51, 68, 85, 102, 136, and 170 ms. In both cases, spectra were processed and peaks integrated with NMRPipe and then fit to a single exponential function with LMquick [49]. A rotational correlation time (τ_c_) was calculated from the average T_1_/T_2_ ratio [50]. From the correlation time, a molecular weight was estimated according to the linear relationship τ_c_ = MW * 0.433 + 0.775 published at the University of San Diego NMR Center (http://sopnmr.ucsd.edu/biomol-tools.htm).

### X-ray crystallography

Crystals of selenomethionine labeled CASKIN2 SAM1-SAM2 were obtained by hanging drop vapor diffusion at 4 °C with equal parts of a 0.6 mM protein solution in T300 buffer and reservoir solution containing 0.1 M Tris pH 7.5, 2.4 M sodium formate, 5 mM DTT. After 24 h, mature crystals were cryoprotected with the same crystallization solution containing 15 % glycerol and flash frozen in liquid nitrogen prior to diffraction experiments. A diffraction dataset using the single anomalous dispersion method at the peak wavelength was acquired at the Canadian Light Source beam line 08B1-1 with a Rayonix MH300HE area detector [51]. All data were processed using XDS [52]. The calculated Matthews coefficient [53] of 4.43 Å^3^/Da suggested the presence of one molecule in the asymmetric unit leading to a solvent content of 72 %. Phasing, density improvement, solvent flattening and refinement was performed with Phenix [54]. Six selenium sites were identified and an initial model was produced with AutoSol. From it, a partial model containing 113 of 166 amino acids was achieved with AutoBuild. This model was completed by successive cycles of refinement using Phenix-Refine and manual rebuilding in Coot [55]. Rigid body refinement and secondary structure restraints were applied throughout the refinement process. In the final refinement stages, target weight optimization was performed. No water molecules were added. Structural analysis was performed with MolProbity [56] and PROCHECK [57]. Backbone RMSD was calculated with SSM [58].

## Additional file

Additional file 1: Figure S1. A composite 2mFo-DFc omit map of the CASKIN2 SAM domain tandem linker region. **Figure S2.** Comparison of wild type and double mutant SAM tandem proteins by NMR spectroscopy. PDF 420 kb

## Abbreviations

CASKIN: CASK interacting protein
NMR: Nuclear magnetic resonance
PTM: Post-translational modification
SAM: Sterile alpha motif domain
