# Supplementary material for: A new mode of SAM domain mediated oligomerization observed in the CASKIN2 neuronal scaffolding protein
Source: Cell Commun Signal. 2016 Aug 22;14(1):17. doi: 10.1186/s12964-016-0140-3 (PMC4994250; doi:10.1186/s12964-016-0140-3)
Supplement: Additional file 1: Figure S1. — A composite 2mFo-DFc omit map of the CASKIN2 SAM domain tandem linker region. Figure S2. Comparison of wild type and double mutant SAM tandem proteins by NMR spectroscopy. PDF 420 kb [file 12964_2016_140_MOESM1_ESM.pdf]

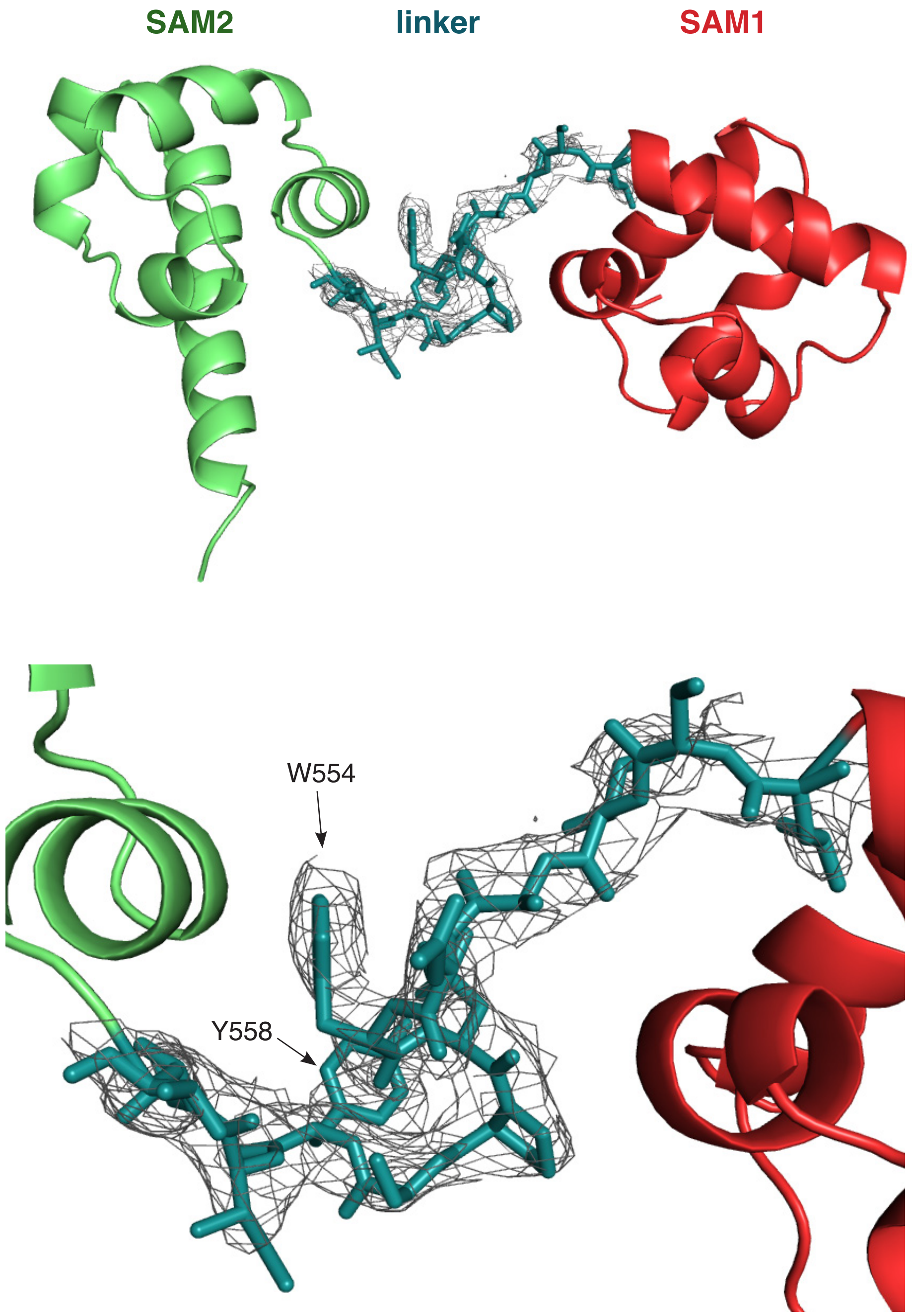

Supplementary Fig S1 — Omit map of the CASKIN2 SAM domain tandem linker region. A composite 2mFo-DFc omit map covering the linker (549-561) was calculated with Phenix 1.10.1 (iterative removal of phase bias simulated annealing for low resolution structures). The map shown was contoured at 1.0σ. The SAM domains and the linker are colored separately for reference. Selected aromatic amino acids are labeled for reference.

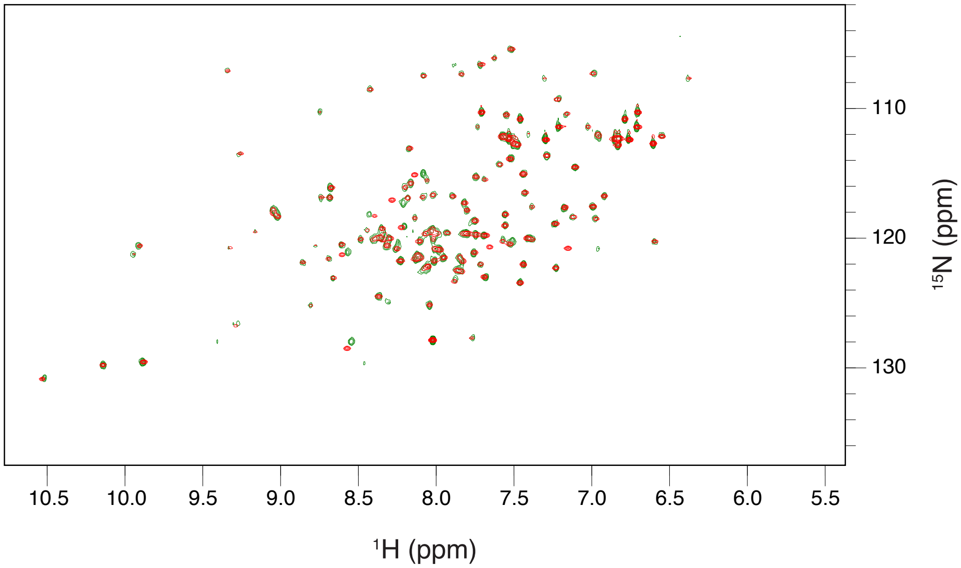

Supplementary Fig S2 — Comparison of wild type and double mutant SAM tandem proteins by NMR spectroscopy. Overlay of ^{1}H-^{15}N HSQC spectra acquired at 298 K of the wild type protein (green; 10 μM, 20 mM sodium phosphate pH 7.8, 300 mM NaCl) and G537D/K540E double mutant (red; 800 μM, 20 mM sodium phosphate pH 7.8, 150 mM NaCl).
